# Using decision trees to examine risk profiles for cannabis use among large samples of underage youth before and after cannabis legalization in Canada

**DOI:** 10.1016/j.abrep.2025.100632

**Published:** 2025-10-10

**Authors:** Scott T. Leatherdale, Katelyn Battista, Karen A Patte, James MacKillop, Richard Bélanger

**Affiliations:** a School of Public Health Sciences, University of Waterloo, Waterloo, Canada; b Department of Health Sciences, Faculty of Applied Health Sciences, Brock University, St. Catherines, Canada; c Peter Boris Centre for Addictions Research, St. Joseph’s Healthcare Hamilton and McMaster University, Canada; d Michael G. DeGroote Centre for Medicinal Cannabis Research, McMaster University and St. Joseph’s Healthcare Hamilton, Canada; e VITAM - Centre de recherche en santé durable, Université Laval, Quebec City, Canada; f Department of Pediatrics, Faculty of Medicine, Université Laval, Quebec City, Canada

**Keywords:** Adolescent/youth, Cannabis legalization, Cannabis use, Decision tree, Mental health

## Abstract

**Background:**

This paper compares risk profiles for cannabis use among large samples of youth in the school years preceding (2017–18, T_1_) and four years following (2021–22, T_2_) cannabis legalization in Canada.

**Methods:**

COMPASS Study data from students across 85 secondary schools that participated in both the T_1_ and T_2_ waves were used. A novel classification tree approach examined current cannabis use (past 30-day), modelling complex interactions among multiple risk factors simultaneously in the T_1_ and T_2_ samples.

**Results:**

At T_1_, 15.0 % of students reported current cannabis use, compared to 12.3 % of students at T_2_. The classification tree at T_1_ identified six unique risk profiles. The highest risk group (Pr = 0.269) was large (30.4 % of the sample) and comprised students who placed lower value on getting good grades and spent 45 min or more per day texting. The classification tree at T_2_ identified 11 unique risk profiles. The highest risk group (Pr = 0.27) was large (18.8 % of the sample) and comprised students who again placed lower value on getting good grades but also reported not eating breakfast daily and having elevated anxiety.

**Discussion:**

Cannabis never use increased and current cannabis use slightly decreased among underage youth in a 4-year period spanning cannabis legalization. The relative importance ranking of risk factors for predicting current cannabis use changed considerably from T_1_ to T_2_. This suggests that prevention efforts need to adapt over time to target the relevant risk factors associated with cannabis use.

## Highlights

• Cannabis never use increased in a 4-year period spanning cannabis legalization.
• Current cannabis use decreased in a 4-year period spanning cannabis legalization.
• Risk factors for current cannabis use changed from pre- to post-legalization.
• Internalizing mental health conditions were important risk factors post-legalization.

## 1 Introduction

Cannabis use among youth is higher in Canada than most other countries globally (Office of Research and Surveillance., 2017, UNICEF Office of Research, 2013). In October 2018, the Canadian government legalized non-medical cannabis use for adults via the Cannabis Act (Department of Justice, 2018). Prospective evidence evaluating the short-term impact of cannabis legalization on underage youth cannabis use suggest that one-year post legalization there was no significant difference in cannabis use trends among youth since legalization (Zuckermann et al., 2021), although the prevalence of occasional cannabis use did decline substantially pre- to post-legalization (19.8 % to 10.4 %) (Rotermann, 2020). Examination of medium-term trends is complicated by the confounding influence of the COVID-19 pandemic (World Health Organization, 2023), which was shown to be associated with reduced adolescent cannabis use rates (Dumas et al., 2020, Leatherdale et al., 2021). This decline in rates may be partially attributed to increased parental supervision and reduced access during lockdowns (Dumas, Ellis & Litt, 2020), with corresponding potential for a post-pandemic renormalization to pre-pandemic rates of use (Battista et al., 2025). Given the confounding influence of COVID-19, prevalence rates alone do not provide an adequate picture of medium-term changes in youth cannabis use post-legalization.

Clearly understanding the impacts of cannabis legalization (and COVID-19) on cannabis use prevalence rates can be difficult to robustly disentangle given limitations in the data available (e.g., no unexposed control group data are available). However, novel insight on the potential impacts can still be gained by identifying changes in the risk profiles associated with cannabis use over time (i.e., what are the risk profiles of youth who are using cannabis after legalization compared to before legalization) as legalization has likely altered social norms surrounding cannabis. For instance, trend studies have found changes in adolescent perceptions surrounding cannabis post-legalization that include perceived easier access (Fischer et al., 2021, Nguyen et al., 2023), and changes in perceptions of harm (Fischer et al., 2021). These changes in social norms surrounding cannabis may lead to changes in sociodemographic, psychosocial, and/or behavioural risk profiles of youth most likely to use cannabis. If the risk factors for cannabis use among youth have changed over time, this would provide valuable insight to inform ongoing cannabis control efforts.

To date, published studies have not directly examined changes in behavioural and psychosocial drivers of youth cannabis use before and after legalization within the Canadian context. As such, it is important to characterize these changes in two large samples of youth surrounding legalization. For instance, a review of pre-legalization studies identified behavioural factors such as truancy and academic performance, as well as psychosocial factors such as bullying, peer influence and school connection (Butler, Romano & Leatherdale, 2022) being associated with cannabis use. Psychosocial wellbeing (Capaldi, Varin & Dopko, 2021) and internalizing psychological disorders (Bolanis et al., 2020) are also associated with cannabis use, though causal direction is unclear. Additionally, previous research in the school-year preceding legalization (2017) found that screen time, sleep, and use of other substances to be important behavioural risk factors along with psychosocial wellbeing, emotion dysregulation, and depressive symptoms (Romano et al., 2019). To properly examine changing youth risk profiles for post-legalization cannabis use, it is important to understand the complex interactions among these various sociodemographic, behavioural, and psychosocial factors.

While available trend studies provide some sociodemographic breakdowns, these studies typically do not also simultaneously examine behavioural or psychosocial correlates and rarely account for interacting influences. Moreover, broader contextual factor that may influence cannabis use behaviour (e.g., income inequality in the communities in which youth live) have not been explored. As such, the objective of the current study is to fill this literature gap by comparing adolescent risk profiles for cannabis use among large samples of youth in the school years preceding (2017–18) and four years following (2021–22) cannabis legalization in Canada. We used a novel classification tree approach (Breiman, Friedman, Olshen & Stone, 1984) that models complex interactions among multiple risk factors simultaneously using a decision tree structure. This allowed us to simultaneously compare the relative influence of a wide range of sociodemographic, behavioural, and psychosocial factors on current cannabis use, and then identify profiles for highest risk groups before and after legalization.

## 2 Methods

### 2.1 Study and sample

The COMPASS study is an ongoing prospective cohort study (2012–2027) of Canadian secondary school students in Ontario, Alberta, British Columbia, Quebec, and Prince Edward Island. COMPASS annually collects student- and school-level data related to healthy eating, physical activity, sedentary behaviour, substance use, mental health, bullying, school connectedness, and academic achievement. COMPASS has received ethics clearance from the University of Waterloo Research Ethics Board (ORE 30118) and all participating school boards. Additional details about the COMPASS host study are available in print (Leatherdale et al., 2014) and online (https://uwaterloo.ca/compass-system).

COMPASS uses purposeful sampling to recruit whole-school samples based on their use of active-information, passive-consent parental permission protocols. The current study uses student-level data from students in grades 9–12 (Secondary 3–5 in Quebec) across 85 schools that participated in both the 2017–18 (*pre-legalization, T_1_*) and 2021–22 (*post-legalization, T_2_*) waves of COMPASS. The T_1_ sample includes n_1_ = 38,885 students (participation rate 81.7 %) and the T_2_ sample includes n_2_ = 29,619 students (participation rate 61.6 %).

### 2.2 Measures

Student-level data are collected using an anonymous, self-administered questionnaire. At T_1_, a machine-readable paper-based questionnaire was administered during scheduled class time, while at T_2_, the same questionnaire and questions on cannabis use were administered using a secure online platform. Further details on questionnaire administration and protocol changes due to COVID-19 are available (Reel, Battista & Leatherdale, 2020). The current study examined an outcome measure of cannabis use frequency as well as 31 variables that were measured in both the 2017–18 and 2021–22 versions of the COMPASS student-level questionnaire.

#### 2.2.1 Cannabis use

To assess cannabis use, students were asked “In the last 12 months, how often did you use marijuana or cannabis? (a joint, pot, weed, hash)”, with response options ranging from “I have never used marijuana” or “I have used marijuana but not in the last 12 months” to “Every day”. Students were classified as *current users* if they indicated use “once a month” or more frequently.

### 2.3 Demographic, Psychosocial, Behavioural, and Mental Health Indicators

Sociodemographic measures include student age, sex, ethnicity, weekly spending money, Body Mass Index (BMI) classification based on reported height and weight, and weight self-description. Behavioural measures include weight loss intention, average daily time in moderate-to-vigorous physical activity, days of strength training per week, participation in school intramural sports, participation in varsity sports, participation in out-of-school league sports, daily breakfast consumption, average daily sleep time, and average daily time spent watching TV or movies, playing video games, surfing the internet, texting/messaging, and doing homework. Psychosocial measures include perceptions of a happy home life, parental expectations, the ability to talk about problems with family, the ability to talk about problems with friends, being bullied, school connectedness, importance of good grades, and future education aspirations. Mental health measures included anxiety (GAD-7) (Spitzer et al., 2006), depression (CESD-10) (Andresen et al., 1994), emotion regulation (DERS) (Neumann et al., 2010), and flourishing (Diener et al., 2009).

### 2.4 Analyses

Students with missing data on the cannabis use measure were removed, resulting in final analytic samples of n_1_ = 38,334 at T_1_ and n_2_ = 27,079 at T_2_. Descriptive statistics for these samples are provided (refer to Supplementary Table A). Descriptive statistics for sample demographic characteristics, frequency of cannabis use, and rates of current cannabis use were calculated for both T_1_ and T_2_.

In order to examine the distinct pre- and post-legalization risk profiles for cannabis use, separate classification trees predicting current use of cannabis were constructed using CART for the T_1_ and T_2_ samples. For interpretation, the final tree nodes with cannabis current use rates over 5 % higher than the sample average (root node) were classified as high-risk groups. To rank and compare key risk factors for cannabis use between T_1_ and T_2_, relative variable importance (rVI) was calculated for each covariate based on its contribution to tree model fit as a primary or competitor branching variable.

All covariates were included as predictors in each classification model and missing values were accounted for using surrogate splitting variables (Therneau & Atkinson, 2022a). Our choice of specific parameter inputs were made based on preliminary testing to ensure validity and stability within our sample. In particular, our use of a weighted loss function in proportion to the class imbalance improves the sensitivity of our decision tree models to identify cannabis users. Due to class imbalance in rates of cannabis current use, a weighted loss function proportional to the class imbalance was used to improve model sensitivity. The Gini-index was used to measure node impurity for splitting, and tree depth was capped at four levels of splits to avoid model over-complexity given the large number of covariates examined. Area under the receiver operating characteristic curve (AUC) was used as the criterion for final tree selection, with pruning performed to mitigate overfitting using 10-fold cross-validation to select the smallest tree having an AUC within one standard error of the maximum AUC (i.e., the “1-SE” rule). The “rpart” (Therneau, Atkinson & Ripley, 2022b) routine within the “caret” (Kuhn et al., 2023) package was used in R software version 4.3.0 (R Core Team, 2023).

## 3 Results

Demographic characteristics and cannabis use frequencies for the T_1_ and T_2_ student samples are provided in Table 1. Additional descriptive statistics for the additional covariates in the models are provided in Supplementary Table A at the end of the manuscript. As shown, when compared to the T_1_ pre-legalization sample, the T_2_ post-legalization sample had a slightly higher proportion of females (53.0 % vs. 50.9 %) and minority ethnicity students (34.9 % vs. 33.2 %), while average age was the same across samples at 15.6 years. At T_1_, 15.0 % of students were current cannabis users, whereas 12.3 % of students were current cannabis users at T_2_.

**Table 1:** Demographic characteristics and frequencies of cannabis use in the 2017–18 (pre-cannabis legalization) and 2021–22 (post-cannabis legalization) COMPASS samples.

**Sample Characteristics**	**2017**–**18 (T_1_)**	**2021**–**22 (T_2_)**
	*Pre-Cannabis Legalization*	*Post-Cannabis Legalization*
	(*n = 38,334*)	(*n = 27,079*)
	**N (%)**	**N (%)**
**Sex**		
Female	19,416 (50.9)	13,982 (52.9)
Male	18,718 (49.1)	12,452 (47.1)
[missing]	200	645

**Ethnicity**		
White	25,476 (66.7)	17,180 (64.7)
Asian	4,339 (11.4)	3,121 (11.7)
Multiethnic	3,132 (8.2)	2,128 (8.0)
Other	2,678 (7.0)	2,187 (8.2)
Black	1,592 (4.2)	1,198 (4.5)
Latino	996 (2.6)	755 (2.8)
[missing]	121	510

**Cannabis Use Frequency**		
Never	26,796 (69.9)	20,245 (74.8)
Not in the past year	1,862 (4.9)	1,295 (4.8)
Less than once a month	3,917 (10.2)	2,196 (8.1)
Once a month	1,090 (2.8)	583 (2.2)
2 to 3 times a month	1,379 (3.6)	690 (2.5)
Once a week	596 (1.6)	306 (1.1)
2 to 3 times a week	804 (2.1)	390 (1.4)
4 to 6 times a week	619 (1.6)	394 (1.5)
Everyday	1,271 (3.3)	980 (3.6)

**Current Cannabis Use**		
No	32,575 (85.0)	23,736 (87.7)
Yes	5,759 (15.0)	3,343 (12.3)

**Age** (in years)		
Mean (sd)	15.6 (1.2)	15.6 (1.1)
[missing]	36	25

### 3.1 Relative importance Rankings of risk factors for current cannabis use

The relative variable importance (rVI) ratings of the model-ranked predictors of current cannabis use at T_1_ and T_2_ are shown in Table 2. Many risk factors were common across years, though the relative ranking of risk factors changed considerably from pre- to post-legalization. The top predictors of current cannabis use at T_1_ were time spent texting/messaging (rVI 100), daily breakfast consumption (rVI 100), and time spent doing homework (rVI 96); all of which also remained as important predictors in 2021–22 (rVI’s ≥ 29). The top predictors of current cannabis use at T_2_ were depression (rVI 100), happy home life (rVI 86; which increased considerably in importance from 2017 to 18), and students believing that getting good grades was important (rVI 97; which remained an important predictor of cannabis use in 2021–22). Several other factors were identified as important predictors both pre- and post-legalization: school connectedness (rVI 60 and 14), post-secondary educational aspirations (rVI 41 and 4), weekly spending money (rVI 23 and 27), age (rVI 23 and 53), and ethnicity (rVI 5 and 36). Time spent surfing the internet was ranked as an important risk factor at T_1_ (rVI 5) but not at T_2_. Additionally, several new risk factors emerged post-legalization: anxiety (rVI 54), difficulty in emotion regulation (rVI 64), being bullied in past 30 days (rVI 19), flourishing (rVI 11), and weight loss intentions (rVI 3).

**Table 2:** Relative variable importance rating of model-ranked^†^ predictors of current cannabis use in the 2017–18 (pre-cannabis legalization) and 2021–22 (post-cannabis legalization) COMPASS samples.

**2017**–**18**		**2021**–**22**	
*Pre-Cannabis Legalization (n = 38,334)*		*Post-Cannabis Legalization (n = 27,079)*	
**Relative Variable Importance (rVI)**		**Relative Variable Importance (rVI)**	
**Characteristic**	**rVI%**	**Characteristic**	**rVI%**
Time Texting (min/day)	100	Depression (CESD-10)	100
Eat Breakfast Daily	100	Getting Good Grades is Important	97
Time Spent Doing Homework (min/day)	96	Happy Home Life	86
Getting Good Grades is Important	74	Emotional Regulation (DERS)	64
School Connectedness	60	Eat Breakfast Daily	58
Education Aspirations After High School	41	Anxiety (GAD-7)	54
Depression (CESD-10)	30	Age	53
Weekly Spending Money	23	Time Spent Doing Homework (min/day)	53
Age	23	Ethnicity	36
Time Surfing the Internet (min/day)	5	Time Texting (min/day)	29
Ethnicity	5	Weekly Spending Money	27
Happy Home Life	5	Bullied in past 30 days	19
		School Connectedness	14
		Flourishing	11
		Education Aspirations After High School	4
		Weight Loss Intentions	3

*Note*^†^ Predictors that were not used as either primary or competitor splits in the classification tree models received a model relative variable importance score of 0 and are not included in the above table.

It is also important to highlight that several factors examined in these models did not emerge as important risk factors for current cannabis use in either the T_1_ or T_2_ samples: sex, weight status (based on BMI classification), weight perception, moderate-to-vigorous physical activity, training, participation in school intramural sports, participation in varsity sports, participation in community sports, daily sleep time, daily time spent watching TV or movies, daily time spent playing video games, parental expectations, the ability to talk about problems with family, and the ability to talk about problems with friends.

### 3.2 Risk profiles for current cannabis use

**Fig. 1:**
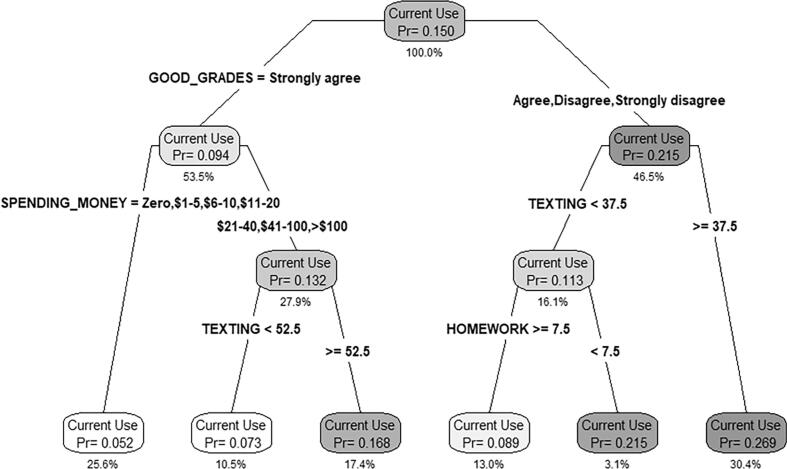
CART-based classification tree for current cannabis use among students in the 2017–18 (T_1_, pre-cannabis legalization) COMPASS sample. Notes: Pr = probability of current cannabis use in the group. % = % of the total sample (n_1_ = 38,334).

The classification tree for current cannabis use in the T_1_ sample is shown in Fig. 1. The model identified six unique risk profiles associated with underage youth being current cannabis users. The lowest risk group with a probability of current cannabis use rate (Pr) of 0.052 (i.e., only 5.2 % of youth in this group reported current cannabis use), was large (25.6 % of the sample) and comprised students who strongly valued getting good grades and had under $20 per week in available spending money. Conversely, two high-risk groups emerged, where they are considered high-risk given the Pr for current cannabis use rates in these groups were more than 5 % higher than the overall sample average for T_1_ (where 15.0 % of students reported current cannabis use). The highest risk group (Pr = 0.269) was large (30.4 % of the sample) and comprised students who placed lower value on getting good grades and spent 45 min or more per day texting; more than a quarter of students in this group reported current cannabis use. The second highest risk group (Pr = 0.215) was similar but smaller (3.1 % of the sample), and comprised similar students who placed lower value on getting good grades and spent less than 45 min per day texting/messaging, but also spent no time doing homework.

**Fig. 2:**
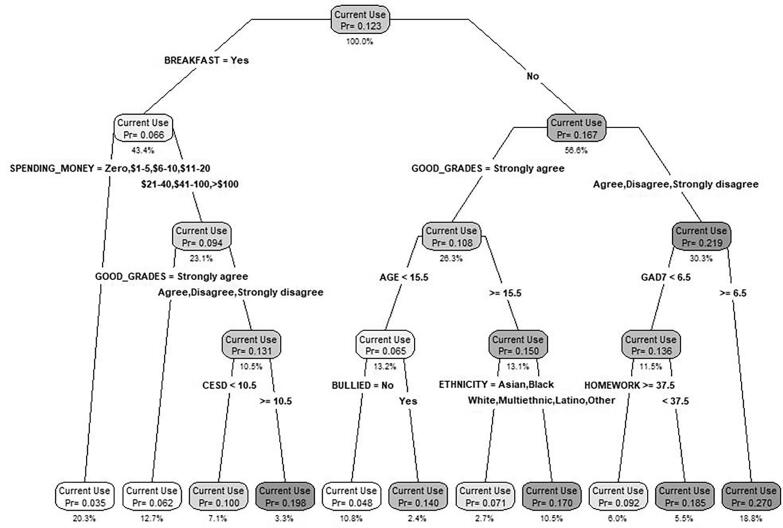
CART-based classification tree for current cannabis use among students in the 2021–22 (T_2_, post-cannabis legalization) COMPASS sample. Notes: Pr = probability of current cannabis use in the group. % = % of the total sample (n_2_ = 27,079).

The classification tree for current cannabis use in the T_2_ sample is shown in Fig. 2. The model identified 11 unique risk profiles associated with underage youth reporting current cannabis use. The lowest risk group (Pr = 0.035) was large (20.3 % of the sample) and comprised students who ate breakfast daily and had under $20 per week in available spending money. Three high-risk groups emerged, where they are considered high-risk given the Pr for current cannabis use rates in these groups were more than 5 % higher than the overall sample average for T_2_ (where 12.3 % of students reported current cannabis use). The highest risk group (Pr 0.27) was large (18.8 % of the sample) and comprised students who did not eat breakfast daily, placed lower value on getting good grades, and had anxiety (GAD-7) scores over 6.5. A second high-risk group (Pr 0.185) was similar but smaller (5.5 % of the sample) and comprised students who did not eat breakfast daily, placed lower value on getting good grades, and had anxiety (GAD-7) scores under 6.5 but also spent 15 min or fewer on homework daily. The third high-risk group (Pr 0.198) was small (3.3 % of the sample) and comprised students who ate breakfast daily, had over $20 per week in available spending money, placed low value on getting good grades, and had depression (CESD-10) scores over 10.5.

## 4 Discussion

This manuscript used the novel methodological approach of classification trees to explore distinct pre- and post-legalization risk profiles for cannabis use in a large sample of youth. Previous research has shown that classification trees are superior to logistic regression with respect to identifying correlates of cannabis use (Dell et al., 2022). Our results highlight an increase in reports of cannabis never use and a slight decline in current cannabis use in our samples. While this is contrary to evidence of a plateau in cannabis use among youth during the early stages of the COVID-19 pandemic (Leatherdale et al., 2021), it may indicate that the declines observed here (a few years out from the pandemic-related restrictions) may not be a result of the pandemic but more likely due to regulations association with legalization and/or changes in social norms. That being said, the more novel finding is that our classification trees identified how the relative ranking of risk factors for predicting current cannabis use changed considerably from pre- to post-legalization. It does appear that there is a meaningful shift in both cannabis use and the predictors of use among youth that occurred over this period of time.

As stated, while many risk factors were common across years, there were also substantial changes in the risk factors identified. These results presented clearly identify that the risk profiles for current cannabis use among youth are not fixed in stone but rather more fluid; it is reasonable to assume they are constantly changing as social norms change over time. As such, consistent with previous cannabis control recommendations (Wellman et al., 2023), prevention efforts may benefit from also adapting over time to target the relevant risk factors associated with cannabis use. Ongoing and timely surveillance efforts are necessary to inform leverage points at a particular period of time.

These results provide robust new evidence that mental health related variables examined have increasing importance with respect to current cannabis use post-legalization relative to pre-legalization. For instance, in the pre-legalization model, the only mental health-related variable with a meaningful relative variable importance score was depression. Whereas, in the post-legalization model, depression had the largest relative variable importance score, followed by the emergent mental health factors of emotional regulation and anxiety. Moreover, in the pre-legalization classification tree (Fig. 1), there were no mental health factors associated with the high-risk groups for current cannabis use. However, in the post-legalization classification tree, both depression and anxiety are characteristics associated with high-risk current cannabis use groups. While this finding is novel, it does align with research suggesting that during this same period of time (where there was also the onset of the COVID-19 pandemic in 2020), population-level changes in mental health occurred (Statistics Canada, 2020). Evidence suggests Canadian students with poor mental health commonly reported using cannabis as a coping mechanism for dealing with the societal changes associated with the early stages of the COVID-19 pandemic (Romano et al., 2021). This is also supported by evidence showing that the COVID-19 pandemic did not have an impact on rates of youth cannabis use among Canadian youth (Leatherdale et al., 2021). As such, it appears that mental health risk factors during the post-legalization period became more important contributors to cannabis use.

Unlike the emergence of mental health variables, there are some characteristics identified in both models as being important correlates of current cannabis use over both time periods examined. Indicators of academic disengagement, such as low value on getting good grades, minimal time spent on homework, lack of school connectedness, and low educational aspirations following high school were key risk factors for cannabis use pre-legalization and remain important post-legalization. While the results suggest their relative importance has decreased somewhat pre- to post-legalization (possibly due to the emergence of the mental health variables within the post-legalization sample), they remain important. This is consistent with research showing that school factors, such as school connectedness, can impact the likelihood of cannabis use. For instance, students with higher school connectedness consistently have a lower likelihood for current cannabis use (Wellman et al., 2023, Varatharajan et al., 2024, Clements-Nolle et al., 2022, Matteau-Pelletier et al., 2020). Evidence also suggests that students with worse than average school performance are more likely to use cannabis (Wellman et al., 2023, Gerra et al., 2020), whereas students who report feeling supported at school are less apt to use cannabis (Moore et al., 2018). While we are unable to determine causality in this study, for schools who want to take action, our results suggest that school-based efforts to improve modifiable risk factors associated with the school context (such as school connectedness, educational aspirations, and valuing homework) among the student population may be effective for reducing youth cannabis use and warrant evaluation.

In terms of sociodemographic predictors, we only observed differences by age and ethnicity among certain subgroups post-legalization. Interestingly, we identified that there were no differences by sex either before or after legalization, while this differs from past research showing that males are more likely to use cannabis then females (Leatherdale et al., 2021, Butler et al., 2023), within the Canadian context post cannabis legalization, there appears to be a narrowing of sex differences in cannabis use among youth (Varatharajan et al., 2024). Moving forward, prevention efforts should be designed to address the needs of both male and female youth.

Key strengths of this study include the very large population-based samples, and the availability of pre- and pot-legalization data. While the COMPASS study is based on self-reported data, which can be prone to recall and social desirability bias, it uses passive consent which is essential for producing robust results that limit self-selection and response bias, particularly for measures of substance use behaviours (Rojas et al., 2008, White et al., 2004). Critically, student names are not required, helping to preserve anonymity and maximally accurate reporting. Possible limitations include COMPASS being based on a convenience sample of participating schools, so the results may not be generalizable to all Canadian youth. However, research has shown that the COMPASS data are comparable to nationally representative data from the Health-Behaviour of School-aged Children Survey (HBSC) (Butler et al., 2023), especially with respect to cannabis use. Moreover, this manuscript did not explore co-occurring substance use as it is already well established that students who use other substances (e.g., alcohol, cigarettes) are substantially more likely to use cannabis (Wellman et al., 2023, Varatharajan et al., 2024). COMPASS does not include measures of peer or parental cannabis use which is an important risk factor for individual cannabis use (Moore et al., 2018). These data also span the early stages of the COVID-19 pandemic, which was previously shown to impact youth mental health and substance use (Romano et al., 2021); however, since research has also shown that the COVID-19 pandemic did not have a substantive impact on rates of youth cannabis use among Canadian youth (even when taking into consideration the transition from paper-based to online survey implementation over the study period) (Leatherdale et al., 2021), it seems reasonable to assume the changes in cannabis use post-legalization are not directly attributable to COVID-19 pandemic impacts. Finally, as with any statistical model, decision trees are subject to limitations, one being the tendency to overfit to sample data relative to other machine learning approaches such as random forests. We partially mitigate this through methodological choices such as employing cross-validation to prune trees and capping the number of levels within this manuscript.

## 5 Conclusion

Given that cannabis use among youth remains common, there is a pressing need to identify the characteristics of youth who are at the greatest risk for cannabis misuse and, in turn, to develop and deploy early prevention and intervention programs tailored to these needs in high-risk youth. This study provides evidence that, in a relatively short 4-year period spanning the cannabis pre-legalization to post-legalization time periods, adolescent cannabis use has declined, but the risk factor profile for cannabis use has substantively changed, increasingly implicating elevations in internalizing mental health conditions. Locally relevant and timely ongoing surveillance efforts are required to inform cannabis control efforts moving forward.

## CRediT authorship contribution statement

**Scott T. Leatherdale:** Writing – original draft, Resources, Methodology, Funding acquisition, Data curation, Conceptualization. **Katelyn Battista:** Writing – review & editing, Visualization, Methodology, Formal analysis. **Karen A Patte:** Writing – review & editing. **James MacKillop:** Writing – review & editing. **Richard Bélanger:** Writing – review & editing, Funding acquisition.

## Declaration of competing interest

The authors declare that they have no known competing financial interests or personal relationships that could have appeared to influence the work reported in this paper.

## Data Availability

Data can be requested at the following link:https://uwaterloo.ca/compass-system/sites/default/files/uploads/documents/compass-data-use-application-form_feb-18-2025.pdf
