# Supplementary material for: Using decision trees to examine risk profiles for cannabis use among large samples of underage youth before and after cannabis legalization in Canada
Source: Addict Behav Rep. 2025 Oct 10;22:100632. doi: 10.1016/j.abrep.2025.100632 (PMC12554066; doi:10.1016/j.abrep.2025.100632)
Supplement: Supplementary Data 1 [file mmc1.docx]

**Supplementary Table A** Descriptive characteristics for variables not shown in Table 1 for the 2017-18 (pre-cannabis legalization) and 2021-22 (post-cannabis legalization) COMPASS samples.

| **Sample Characteristics** | **2017-18**  *Pre-Cannabis Legalization*  (*n=38,334*)  **N (%)** | **2021-22**  *Post-Cannabis Legalization*  (*n=27,079*)  **N (%)** |
| --- | --- | --- |
| **Weekly Spending Money**  $0  $1-5  $6-10  $11-20  $21-40  $41-100  >$100  [Missing] | 5,999 (18.5)  1,902 (5.9)  2,432 (7.5)  4,538 (14.0)  4,210 (13.0)  5,290 (16.3)  7,991 (24.7)  5,972 | 5,176 (23.2)  916 (4.1)  1,109 (5.0)  2,297 (10.3)  2,328 (10.4)  3,184 (14.3)  7,279 (32.7)  4,790 |
| **Weight Status (based on Body Mass Index)**  Underweight  Healthy Weight  Overweight  Obese  Not Stated | 630 (1.6)  21,097 (55.0)  4,706 (12.3)  2,273 (5.9)  9,628 (25.1) | 374 (1.4)  12,392 (45.8)  2,497 (9.2)  1,073 (4.0)  10,743 (39.7) |
| **Weight Perception**  Very Underweight  Slightly Underweight  About the right weight  Slightly overweight  Very overweight  [Missing] | 738 (2.0)  5,380 (14.3)  22,328 (59.2)  8,051 (21.3)  1,222 (3.2)  615 | 680 (2.5)  4,241 (15.8)  15,363 (57.1)  5,663 (21.0)  973 (3.6)  159 |
| **Weight Loss Intentions**  Lose weight  Gain weight  Stay the same weight  No intention  [Missing] | 13,177 (34.9)  6,184 (16.4)  7,894 (20.9)  10,543 (27.9)  536 | 9,524 (35.3)  4,794 (17.8)  5,045 (18.7)  7,606 (28.2)  110 |
| **Sleep** (min/day)  Mean (sd)  [missing] | 437.5 (81.9)  2,612 | 479.3 (73.2)  1,262 |
| **Time Watching TV/Movies** (min/day)  Mean (sd)  [missing] | 109.3 (80.2)  904 | 104.5 (83.8)  1,165 |
| **Time Playing Videogames** (min/day)  Mean (sd)  [missing] | 72.2 (92.1)  1,416 | 81.3 (97.6)  1,662 |
| **Time Surfing the Internet** (min/day)  Mean (sd)  [missing] | 107.1 (91.0)  2,038 | 65.5 (79.8)  1,407 |
| **Time Texting** (min/day)  Mean (sd)  [missing] | 86.8 (87.0)  2,163 | 72.5 (76.4)  1,467 |
| **Strength Training** (days/week)  Mean (sd)  [missing] | 2.6 (2.2)  494 | 2.6 (2.3)  535 |
| **Intramural Sports**  Yes  No  None Offered At School  [Missing] | 13,318 (35.2)  23,198 (61.3)  1,338 (3.5)  480 | 8,721 (32.8)  16,832 (63.4)  1,001 (3.8)  525 |
| **Varsity Sports**  Yes  No  None Offered at School  [Missing] | 13,588 (35.8)  23,803 (62.7)  545 (1.4)  398 | 7,959 (29.8)  18,120 (67.9)  596 (2.2)  404 |
| **Community Sports**  Yes  No  None Available  [Missing] | 15,519 (40.9)  21,946 (57.8)  478 (1.3)  391 | 9,049 (33.9)  17,211 (64.5)  439 (1.6)  380 |
| **Moderate-to-Vigorous Physical Activity**  (min/day)  Mean (sd)  [missing] | 104.8 (74.3)  1,168 | 101.0 (74.6)  1,870 |
| **Eat Breakfast Daily**  Yes  No  [Missing] | 17,365 (45.3%)  20,969 (54.7%)  0 | 11,610 (43.5%)  15,107 (56.5%)  362 |
| **Happy Home Life**  Strongly agree  Agree  Neither agree nor disagree  Disagree  Strongly disagree  [Missing] | 15,064 (39.7)  14,605 (38.5)  5,511 (14.5)  2,002 (5.3)  789 (2.1)  363 | 9,692 (36.2)  9,844 (36.8)  4,734 (17.7)  1,721 (6.4)  756 (2.8)  332 |
| **Parents Expect Too Much**  Strongly agree  Agree  Neither agree nor disagree  Disagree  Strongly disagree  [Missing] | 3,797 (10.1)  8,007 (21.2)  14,232 (37.8)  9,096 (24.1)  2,558 (6.8)  644 | 2,961 (11.1)  5,944 (22.3)  9,895 (37.1)  6,100 (22.9)  1,782 (6.7)  397 |
| **Ability to Talk with Family About Problems**  Strongly agree  Agree  Neither agree nor disagree  Disagree  Strongly disagree  [Missing] | 9,299 (24.7)  12,452 (33.1)  7,698 (20.4)  5,167 (13.7)  3,049 (8.1)  669 | 6,233 (23.4)  7,699 (28.9)  5,870 (22.0)  4,040 (15.1)  2,837 (10.6)  400 |
| **Ability to Talk with Friends About Problems**  Strongly agree  Agree  Neither agree nor disagree  Disagree  Strongly disagree  [Missing] | 13,737 (36.3)  14,809 (39.1)  5,751 (15.2)  2,220 (5.9)  1,331 (3.5)  486 | 8,600 (32.3)  9,735 (36.5)  4,969 (18.6)  2,142 (8.0)  1,211 (4.5)  422 |
| **Depression** (CESD-10: 0-30)  Mean (sd)  [missing] | 8.9 (6.1)  5,074 | 10.6 (6.8)  2,129 |
| **Anxiety** (GAD-7: 0-21)  Mean (sd)  [missing] | 6.4 (5.7)  2,550 | 8.4 (6.4)  1,397 |
| **Emotional Regulation** (DERS: 6-30)  Mean (sd)  [missing] | 14.3 (4.8)  2,367 | 15.7 (5.3)  1,363 |
| **Flourishing** (8-40)  Mean (sd)  [missing] | 31.8 (5.7)  1,606 | 30.7 (6.1)  987 |
| **School Connectedness** (6-24)  Mean (sd)  [missing] | 14.8 (3.1)  1,954 | 14.5 (3.1)  1,310 |
| **Bullied in past 30 days**  No  Yes  [missing] | 31,147 (81.3)  7,187 (18.7)  0 | 21,446 (84.9)  3,802 (15.1)  1,831 |
| **Time Spent Doing Homework** (min/day)  Mean (sd)  [missing] | 91.6 (75.5)  636 | 84.1 (76.3)  746 |
| **Getting Good Grades is Important**  Strongly agree  Agree  Disagree  Strongly disagree  [Missing] | 19,987 (54.0)  14,167 (38.2)  2,071 (5.6)  821 (2.2)  1,288 | 13,198 (50.7)  10,150 (39.0)  1,881 (7.2)  788 (3.0)  1,062 |
| **Education Aspirations After High School**  Some High School  High School Diploma  College/Trade  University Bachelor  University Post-Grad  [Missing] | 651 (2.1)  1,577 (5.1)  7,413 (24.2)  7,429 (24.2)  13,574 (44.3)  7,690 | 564 (2.6)  1,536 (7.2)  5,072 (23.8)  5,209 (24.4)  8,931 (41.9)  5,767 |
